# Contrasting patterns of trematode and host snail dispersal across clusters of tropical crater lakes

**DOI:** 10.1186/s40249-026-01488-9

**Published:** 2026-08-24

**Authors:** Cyril Hammoud, Bert Van Bocxlaer, Julius Tumusiime, Dirk Verschuren, Christian Albrecht, Casim Umba Tolo, Tine Huyse

**Affiliations:** 1 Limnology Unit, Department of Biology, Ghent University, 9000 Ghent, Belgium; 2 Department of Biology, Royal Museum for Central Africa, 3080 Tervuren, Belgium; 3 Mixed Research Unit 8198 Evolution-Ecology-Paleontology, French National Centre for Scientific Research, University of Lille, F-59000 Lille, France; 4 Department of Biology, Mbarara University of Science and Technology, P.O. Box 1410 Mbarara, Uganda; 5 Systematics and Biodiversity Laboratory, Department of Animal Ecology and Systematics, Justus Liebig University, D-35392 Giessen, Germany; 6 Laboratory of Biodiversity and Evolutionary Genomics, University of Leuven, B-3000 Leuven, Belgium

**Keywords:** Comparative population genetics, Haplotype network, Host-parasite phylogeography, Next-generation sequencing, Albertine Rift Valley

## Abstract

**Background:**

Comparing the genetic diversity and population structure of parasites and their hosts can improve understanding of their respective methods of dispersal, demographic histories, and factors driving genetic differentiation. In parasite taxa such as trematodes which have both intermediate and final hosts, comparative population-level analyses may also inform how each host influences gene flow among parasite populations. Here we aimed to assess how intermediate and final hosts influence trematode dispersal by examining the population genetic structure of the aquatic snail *Bulinus tropicus* and three trematodes that use it as intermediate host.

**Methods:**

We used high-throughput amplicon sequencing to simultaneously genotype populations of *B. tropicus* in two clusters of crater lakes in western Uganda (823 snails, 15 lakes) and three infecting trematode taxa (*Petasiger* sp., *Echinoparyphium* sp. and Plagiorchioidea sp.). For each taxon, we quantified genetic diversity in three mitochondrial markers and used analyses of molecular variance to test whether populations were genetically differentiated.

**Results:**

Populations of *B. tropicus* were strongly genetically differentiated among individual crater lakes and lake clusters (Φ_ST_ = 0.78), indicating limited inter-lake dispersal. *Petasiger* sp. haplotype diversity was on average twice as high as that of its intermediate snail host and did not display genetic structuring among lakes (Φ_ST_ = 0.01). This parasite can therefore overcome the dispersal barriers encountered by its snail host, likely facilitated by mobile avian hosts, resulting in a single ‘panmictic’ region-wide population. Contrastingly, *Echinoparyphium* trematodes displayed less genetic diversity and significant genetic differentiation among lakes (Φ_ST_ = 0.35), likely due to limited final host mobility. Finally, Plagiorchioidea sp. was restricted to five neighbouring lakes, and a single haplotype was identified in the 217 infected snails analysed, implying recent introduction into the crater lakes.

**Conclusions:**

Contrasting patterns of genetic diversity and population structure among three parasitic trematodes and their common intermediate snail host illustrate how multi-host life cycles mediate dispersal dynamics and generate distinct evolutionary outcomes in hydrographically fragmented aquatic habitats. These findings demonstrate how multi-host life cycles shape parasite connectivity and evolution, and show that comparative population genetics can provide valuable insights into the dispersal ecology of understudied parasite species.

**Supplementary Information:**

The online version contains supplementary material available at 10.1186/s40249-026-01488-9.

## Background

**Fig. 1:**
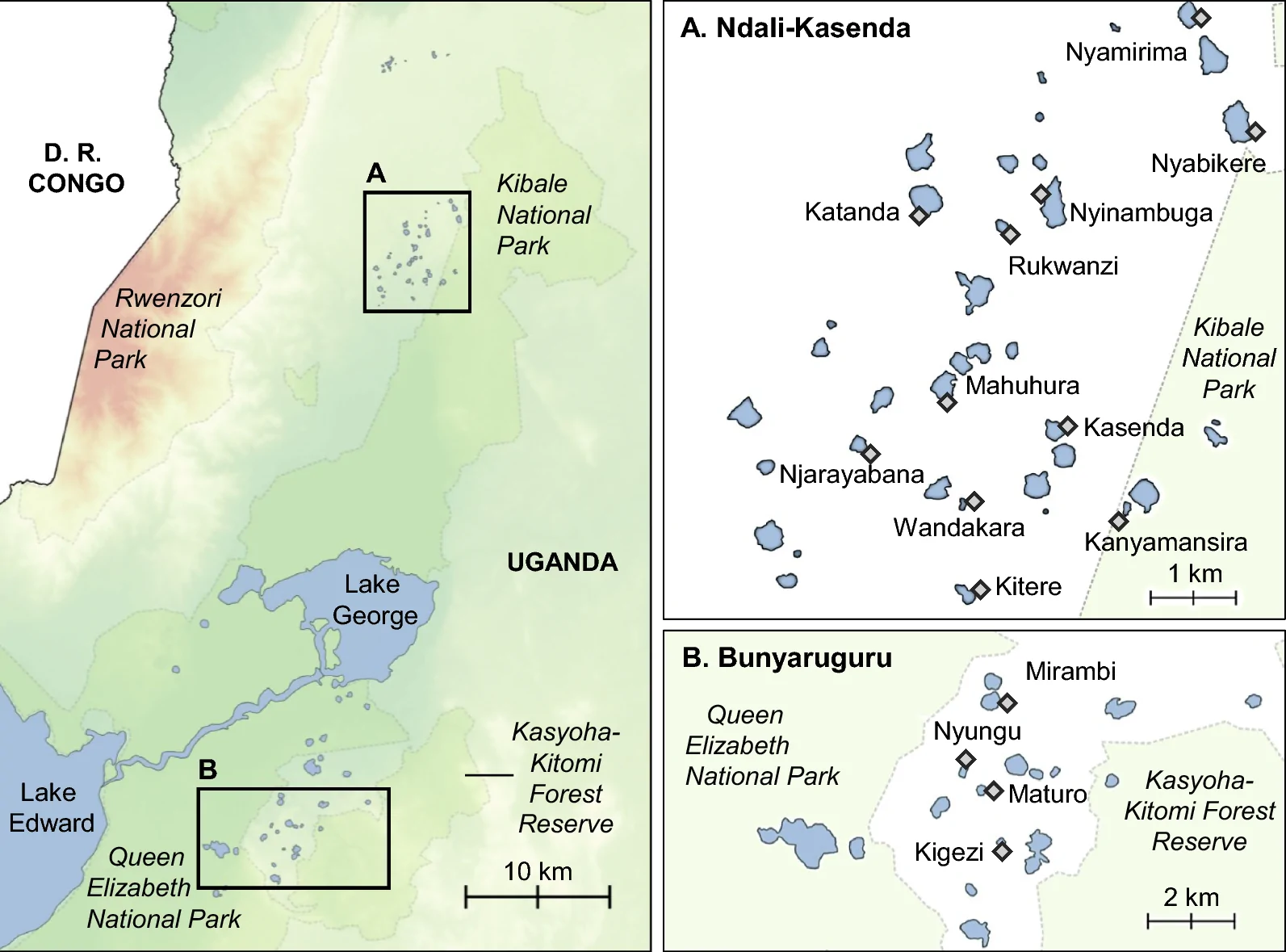
Study region of crater lakes in western Uganda where snail and trematode populations were sampled. The left panel shows the regional topographic setting, the right panel a zoom-in on the two clusters of crater lakes yielding study material. National parks and other protected areas are shaded in green and bounded by stippled lines. Only the 15 study lakes out of the 34 lakes surveyed are labelled by name (coordinates in Table S1). Adapted from [13], using [40] as basemap

Characterising the dispersal patterns of parasites is essential to understand their spatial distribution, ecological preferences, and to predict how anthropogenic influences may affect their future spreading [1, 2]. However, documenting these dispersal patterns can be arduous due to the difficulty of tracking parasites in the environment, especially for parasites with life cycles involving multiple host species (i.e., intermediate and final). Comparative population genetic analyses allow to shed light on the differential dispersal capacities and gene flow among populations of parasites and their hosts [3–5]. In addition, such analyses may also provide insights into the demographic history of parasite and host populations and the microevolutionary processes underlying genetic differentiation (e.g., [5, 6]). In this study, we use high-throughput amplicon sequencing (HTAS) data of multiple mitochondrial markers to perform comparative population genetic studies of three species of parasitic trematodes and their intermediate snail host *Bulinus tropicus* to understand how multi-host life cycles contribute to different relative dispersal capacity, patterns of gene flow, and evolutionary trajectories across 15 hydrographically isolated tropical crater lakes. These lakes are distributed in two distinct lake clusters located ~ 100 km apart (Fig. 1). The exact age of each lake basin is unknown, but geological evidence suggests that they formed between around 50,000 and 8,000 years ago by phreatomagmatic eruptions [7, 8]. Subject of long-lasting limnological investigation (e.g., [8–12]), these lakes are now environmentally well-characterised. Previous surveys of their aquatic mollusc fauna [13–15] indicate that *B. tropicus* is regionally the most common species of aquatic snail hosting parasitic trematodes, and therefore most suited to investigate the relative dispersal capabilities of trematodes and their intermediate host.

*B. tropicus* is a freshwater snail commonly found in standing and slow-flowing water bodies throughout eastern and southern Africa [16]. Like most *Hygrophila* snails (*Pulmonata partim*), it can disperse between water bodies that lack a hydrographic connection, likely via propagules sticking to the plumage of aquatic birds [17]. *B. tropicus* is diploid, hermaphroditic and frequently performs self-fertilisation, which enhances the likelihood to establish new populations [18]. With life spans ranging from a few weeks to 3 months and high fecundity [19], *B. tropicus* and various other *Bulinus* species are typical r-strategists coping well with habitat instability [20]. *B. tropicus* is of veterinary significance as an intermediate host for trematode parasites, including species of *Schistosoma* and *Calicophoron*, which can cause disease in livestock [16]. In the crater lakes of western Uganda, *B. tropicus* populations host diverse trematode communities, many species of which have not yet been formally described [13, 21]. Indeed, the diversity of African trematodes remains largely uncharted, both with regard to representation in molecular databases and information on their ecology (e.g., [22, 23]).

Trematodes are obligate endoparasitic flatworms with a complex life cycle, generally completed within a few months, with a snail as typical (first) intermediate host in which they reproduce asexually, and a broad range of vertebrates as final hosts, in which they reproduce sexually [24]. Many trematode species also use secondary intermediate hosts, in which they do not reproduce but which facilitate transmission to the final host via trophic interactions [25]. Trematodes are diploid, hermaphroditic (except Schistosomatidae, which are dioecious) and some species can self-fertilise [26]. Most trematodes castrate their intermediate snail hosts [24], reducing snail effective population sizes. As self-fertilisation also lowers effective population size, the reproductive mode of both *B. tropicus* and many of its trematode parasites promotes genetic drift, potentially producing strongly structured populations with low genetic diversity [27, 28]. At the same time, the short generation time of both trematodes and host snails may promote high genetic diversity at the population level [29]. Beyond reproductive strategies, genetic diversity and population structure of both snail hosts and trematodes are influenced by the ecology of the species studied and the demographic history of these populations. Aquatic trematodes with autogenic life cycles (i.e. completing their life cycles exclusively with aquatic hosts) tend to display more genetic structure than populations of allogenic species (i.e., those having both aquatic and terrestrial hosts), because the lack of terrestrial hosts hampers dispersal over land [30]. Similarly, populations of trematode species with more intermediate hosts in their life cycles are expected to be genetically less structured, because additional intermediate hosts may increase dispersal opportunities [31] and outcrossing [32].

In this study, we aim to understand how trematode dispersal capacities are impacted by intermediate and final hosts. To do so, we first perform molecular characterization of the intermediate host snail *B. tropicus* and three of its infecting parasites, and then examine geographic patterns of genetic diversity and structure among populations within and across lake clusters.

## Methods

### Study system and material collected

#### Host-parasite systems

The present study involves three trematode species, namely *Petasiger* sp. 5, *Echinoparyphium* sp. and Plagiorchioidea sp. 1 (nomenclature assigned in [13]), which, as verified by shedding experiments [13], use *B. tropicus* as first intermediate host. We focus on these three species as they were found sufficiently abundantly for robust genetic characterization of their populations, enabling comparative population genetic analyses that also include the snail host. By simultaneous genetic characterization of the three parasites and their intermediate host snail, we examine geographic patterns of genetic diversity and structure among lakes and lake clusters to understand how trematode dispersal is impacted by the biology of the intermediate and (putative) final hosts. The three target parasite species were delimited taxonomically by [13, 21], using molecular data to characterize barcoding gaps separating inter- and intra-specific variation using the method of [33]. The three taxa were then identified using the Basic Local Alignment Search Tool (BLAST, [34]) and multi-marker phylogenetic analysis. Here we simplify their nomenclature to *Petasiger* sp., *Echinoparyphium* sp. and Plagiorchioidea sp. This particular *Petasiger* sp. (Echinostomatidae) was molecularly characterized by [35] from cercariae (larval trematodes) shed by several *Bulinus* species in Kenya. The morphology of these cercariae closely resembles those of *Petasiger variospinosus*, which as demonstrated experimentally can use *B. tropicus* as first intermediate host, the African clawed toad (*Xenopus laevi*s) as second intermediate host and the reed cormorant *Microcarbo africanus* as final host [36]. More recently, the same *Petasiger* sp. was also identified from infected cyprinid fish in South Africa [22]. Here, we presume that the *Petagiser* sp. inhabiting our study lakes has a three-host life cycle with in addition to *B. tropicus*, a local fish or amphibian as second intermediate host and piscivorous waterbirds (potentially cormorants) as final host [37, 38]. *Echinoparyphium* sp. (Echinostomatidae) was molecularly described by [35] from a single cercaria shed by *B. tropicus* in Kenya. The only known African *Echinoparyphium* species transmitted by *B. tropicus* is *E. elegans*, which uses this snail as both first and second intermediate host and can infect a variety of final hosts ranging from wild and domesticated birds to small mammals [39]. The *Echinoparyphium* sp. in our study lakes could not be identified with certainty as *E. elegans*, because the ethanol-preserved cercariae from our shedding experiment do not display all diagnostic morphological features needed to confirm conspecificity [35] and public databases lack matching genetic reference sequences [13]. However, if not conspecific it must be closely related, allowing us to assume that the parasite has a metacercarial life stage in either *B. tropicus* or another aquatic animal, and uses birds and/or small mammals as final hosts. The third trematode taxon selected for this study could be taxonomically identified to superfamily level only, due to lack of similar genetic sequences in public databases [13]. Hence the life cycle and final host(s) of our Plagiorchioidea sp. remain unknown.

#### Material collection

For this study, we sampled *B. tropicus* snails and their infecting trematode parasites in February 2019, by surveying 34 freshwater crater lakes located either in- or outside national parks in western Uganda (Fig. 1). The snails were collected by scooping a metal sieve (4 mm mesh size) mounted on a handle through nearshore vegetation and sediments, consistently for ~ 45 min along a ~ 20-m stretch of shoreline in each lake. Recovered snails were sacrificed by heat shock and stored in 80% ethanol for DNA preservation. *B. tropicus* was found in 28 of the surveyed lakes, but for robust genetic characterization of the trematode communities (see [13]) we here focus on the 15 lakes that yielded ≥ 50 *B. tropicus* specimens. Eleven of these 15 lakes belong to the Ndali-Kasenda cluster, which is distributed across highlands forming the eastern flank of the Albertine Rift Valley; the other four lakes belong to the Bunyaruguru cluster located south of Lake George (Tables 1, S1, Fig. 1). All 15 lakes and surrounding crater catchments are located outside of national parks, except for Kanyamansira, which lies inside Kibale National Park with its catchment straddling the park border [8]. As such, all study sites are used by local rural communities for fishing and subsistence farming, including small-scale animal husbandry [11].

**Table 1:** Sequence sample size per marker and lake for *Bulinus tropicus*, *Petasiger* sp., *Echinoparyphium* sp. and Plagiorchioidea sp.

Taxon	Marker	Bunyaruguru				Ndali-Kasenda										
		Nyungu (15)	Maturo (191)	Kigezi (47)	Mirambi (34)	Nyabikere (69)	Kitere (33)	Wandakara (18)	Kasenda (141)	Njarayabana (64)	Mahuhura (57)	Nyamirima (7)	Rukwanzi (20)	Nyinambuga (12)	Katanda (81)	Kanyamansira (34)
*Bulinus tropicus*	COI1	15	191	47	34	69	33	18	141	64	57	7	20	12	81	34
	COI2	15	191	47	34	69	33	18	135	64	54	7	19	12	81	33
	NAD1	14	191	47	34	69	33	18	137	64	57	7	20	12	80	33
*Petasiger* sp.	COI1					1	5	1	18		4	2	9		28	3
	COI2					1	14	1	20		29	5	15		65	21
	NAD1	1				6	14	2	26	1	53	17	33		107	38
	cytb					1	10	1	8		20	5	13		55	15
*Echinoparyphium* sp.	COI2	2				4			1							2
	NAD1	4	38			44			13			3				7
	cytb	4	50			39			13			3				7
Plagiorchioidea sp.	NAD1	7	190	1	18				1							
	cytb	4	189	2	15											

Values provided along lake names represent the number of infected snails analysed using high-throughput amplicon sequencing in that lake. *COI1* cytochrome c oxidase subunit 1 mitochondrial gene fragment, *NAD1* NADH dehydrogenase subunit I gene, *cytb* cytochrome b gene

### Molecular characterisation of snails and trematodes

#### Genotyping strategy

We used a two-step molecular approach to simultaneously genotype *B. tropicus* specimens and their infecting trematodes, therewith producing appropriate molecular data for comparative population genetic analyses. In up to ~ 200 *B. tropicus* specimens per lake, DNA was extracted from all soft tissues using the E.Z.NA Mollusc DNA Kit (OMEGA Bio-tek, Norcross, USA) according to manufacturer instructions and diluted in ultrapure water at a 1:10 concentration. Dried DNA vouchers are stored at the Royal Museum for Central Africa (Tervuren, Belgium) under the code “BE.RMCA.MOL.DNA” with reference numbers 1–2,472. Following DNA extraction, we performed a rapid-diagnostic multiplex polymerase chain reaction (RD-PCR, [41]) to detect trematode infections. Infected snails were subsequently submitted to HTAS [42], amplifying multiple genetic markers of snails and their infecting trematode parasites in a single multiplex PCR reaction. For the snails, we sequenced four genetic markers: one fragment of the internal transcribed spacer 1 (ITS1: 446 bp), two fragments of the cytochrome c oxidase subunit 1 mitochondrial gene (COI1 and COI2: 472 and 331 bp, respectively), and one fragment of the NADH dehydrogenase subunit I gene (NAD1: 493 bp). For trematodes we selected five genetic markers to be as taxonomically versatile as possible: one fragment of the internal transcribed spacer 2 (ITS2: 326–526 bp), two COI fragments (COI1 and COI2: 403 and 340 bp, respectively), one NAD1 fragment (469 bp) and one cytb fragment (378 bp; see [42] for details on marker design and testing).

#### Amplicon sequencing and read processing

The resulting libraries were then indexed and pooled (per ~ 500 snails) for parallel sequencing using the Illumina Miseq v3 platform (300 bp pair-ended). After sequencing, reads were demultiplexed per individual snail and per marker, before performing read quality control and amplicon sequence variants (ASVs) inference using the dada2 pipeline [43]. Finally, in each snail, ASVs with low read support were filtered to avoid including spurious sequences in ASV profiles (e.g., resulting from cross-contamination; for details see [13, 42]). A snail was considered infected by a specific trematode taxon if at least one ASV of that taxon was present in the ASV profile of the snail sample after quality control. Different ASVs for the same marker originating from the same snail individual therefore indicated co-infection, either by different trematode species or by several strains of the same species. Detecting trematode DNA in a snail DNA extract does not by itself imply that the infecting parasite can complete its life cycle using this snail as intermediate host. However, shedding experiments by [13] confirmed that the sampled *B. tropicus* populations shed cercariae of the three parasite species studied here, and that multi-marker profiles inferred from high-throughput sequencing are congruent with the genotyping profiles obtained from single cercariae. For further details on the advantages and drawbacks of the HTAS approach, we refer the reader to [42].

#### Genetic dataset

Considering the small size of all 15 study lakes (Fig. 1), for the purpose of this study, we assume that all snails collected in each lake represent a single host population, and similarly that all sequences of a particular trematode species found in snails from that lake belong to a single parasite population. We used the multi-marker molecular profiles of our 823 *B. tropicus* specimens (average and range [minimum–maximum] sample size *n* per lake = 55 [7–191]), 298 *Petasiger* sp. from 11 lakes (*n* = 27 [1–107]), 109 *Echinoparyphium* sp. from six lakes (*n* = 18 [3–44]) and 217 Plagiorchioidea sp. from five lakes (*n* = 43 [1–190]) (Table 1). Here we limit our analysis to mitochondrial markers (COI1, COI2 and NAD1 totalling 1296 bp for snails; COI1, COI2, NAD1 and cytb totalling ~ 1590 bp for trematodes). Nuclear markers (ITS1 in snails and ITS2 in trematodes) were excluded because high levels of intra-genomic variability in trematodes [42] prevented meaningful analysis of these markers at the population level. GenBank accession numbers of all used sequences are provided in Table S2 and population/marker-specific sample sizes are summarised in Table S3. Variable amplification success of selected mitochondrial markers (see Results) prompted marker-to-marker comparisons for trematode populations, instead of multi-marker analyses.

### Analyses of the genetic diversity and structure of snail and trematode populations

#### Population- and marker-specific genetic diversity

First, we analysed patterns of genetic diversity in the host and parasite populations, acknowledging that all our genetic markers are mitochondrial, and therefore maternally inherited, haploid and non-recombining. We aligned all sequences belonging to the same species and marker in Geneious Prime^®^ 2023.0.1, using MUSCLE [44]. We checked for the presence of STOP codons and indels which would result from reading frame shift in the coding regions, to identify and remove potential nuclear mitochondrial DNA sequences (NUMT, [45]). For snails, COI1, COI2 and NAD1 sequences were concatenated per specimen to enable multi-marker analyses. For this snail multi-marker alignment and each genetic marker for each of the three trematode species, we calculated the number of unique haplotypes, the number of polymorphic sites, the haplotype diversity (probability that two randomly sampled individuals in a population have different haplotypes) and the nucleotide diversity π (average proportion of nucleotides differing between the DNA sequences of all pairs of individuals in a population) in each population using Arlequin 3.5.2.2 [46]. We then used pairwise Wilcoxon tests with Hommel’s corrections [47] for multiple comparisons to investigate potential differences in genetic diversity among markers within taxa, and then among taxa, by comparing common markers. As no genetic diversity was observed within or among populations of Plagiorchioidea sp., we did not perform regression analyses for this taxon. For the three other taxa we first compared both the haplotype and nucleotide diversity among markers by successively performing pairwise Wilcoxon tests on these variables among genetic markers in R 4.1.1 (R Core Team, Vienna, Austria [48]). Then we used pairwise Wilcoxon tests to test whether both haplotype and nucleotide diversity in each marker differed between species, except that differences in genetic diversity between *Echinoparyphium* sp. and the two other taxa could not be assessed for COI1 and COI2 because these markers were not successfully sequenced for this taxon. Finally, we tested whether the genetic variation within each population diverged from expectation under a neutral model of evolution by computing Fu’s *Fs* and Tajima’s *D* tests [49, 50] for each genetic marker in Arlequin, with 10,000 permutations. Only populations with at least five specimens were included in the analyses of genetic diversity and tests of neutrality, because very low sample sizes may affect the results.

#### Population genetic structure

Second, we tested for patterns of genetic structure among populations of each host and parasite species. We started by selecting the most suitable substitution model for each marker-species alignment and for the snail multi-marker alignment based on Akaike Information Criterion values in the model selector of MEGA 11 [51]. We then assessed the genetic population structure of the various marker-species combinations using hierarchical (nested) analyses of molecular variance (AMOVA) in Arlequin with 10,000 permutations, relying on the selected substitution models. In addition to non-hierarchical differentiation among lakes (Φ_ST,_), we investigated genetic differentiation at different geographical scales by testing two hierarchical levels of population differentiation: between crater lake clusters and among lakes within each cluster (fixation index = Φ_CT_ and Φ_SC_ respectively). To this aim, AMOVA was used to calculate the non-hierarchical fixation index Φ_ST_ which reflects the contribution of both lake (population) and lake cluster (grouped populations) to total observed genetic variance, as well as the hierarchical fixation indexes Φ_CT_ (reflecting the contribution of lake clusters to total genetic variance) and Φ_SC_ (reflecting the contribution of individual lakes to total genetic variance). Analysis at the lake-cluster level was only performed when the data allowed (i.e., when the dataset included at least two populations in different clusters). For each marker-species combination, we also performed pairwise AMOVA using 10,000 permutations to compute the pairwise differentiation (pairwise Φ_ST_) among all populations. The patterns of genetic structure were illustrated by constructing Templeton, Crandall and Sing (TCS) haplotype networks [52] using statistical parsimony in PopART v. 1.7 [53].

#### Isolation-by-distance

Finally, we tested whether pairwise genetic differentiation among populations was correlated with pairwise geographic distance. Such a correlation would imply a pattern of isolation-by-distance caused by dispersal limitation [54]. We used non-parametric Mantel tests [55] with 10,000 permutations in Arlequin to examine correlative patterns. AMOVA and isolation-by-distance analyses only included populations with at least five successfully sequenced specimens for a given marker, as proposed by [56].

## Results

### Genetic diversity in snail and trematode populations

Sequencing success was consistently high for the three molecular markers used to genotype *B. tropicus*, which allowed us to consistently build multi-marker profiles for each specimen (> 95% successful sequencing in all three markers; Table S3). In contrast, marked disparities were observed among the markers used to genotype trematodes (Table S3). Indeed, only the NAD1 marker was consistently sequenced in all three trematode species (100% of infections by *Petasiger* sp. and Plagiorchioidea sp.; 99% of *Echinoparyphium* sp. infections). The COI1, COI2 and cytb markers had lower sequencing success for *Petasiger* sp. (successful sequencing in 24%, 57% and 43% of samples, respectively). For *Echinoparyphium* sp., sequencing the COI1 and COI2 markers was not or rarely successful (0% and 8%, respectively), whereas cytb sequences were obtained from all detected infections. Finally, for Plagiorchioidea sp. neither COI1 nor COI2 were obtained but the cytb marker was successfully sequenced from most infections (97%). This data limitation originates from the difficulty of designing phylogenetically versatile primers for trematodes, which in turn relates to the paucity of reference sequences for African trematodes in NCBI databases [23] and the high genetic diversity observed in trematodes and flatworms in general [57]. Simultaneously sequencing multiple markers however, ensures a high likelihood that all infecting trematodes are detected using the HTAS workflow, despite variable success in marker-specific sequencing [42].

**Fig. 2:**
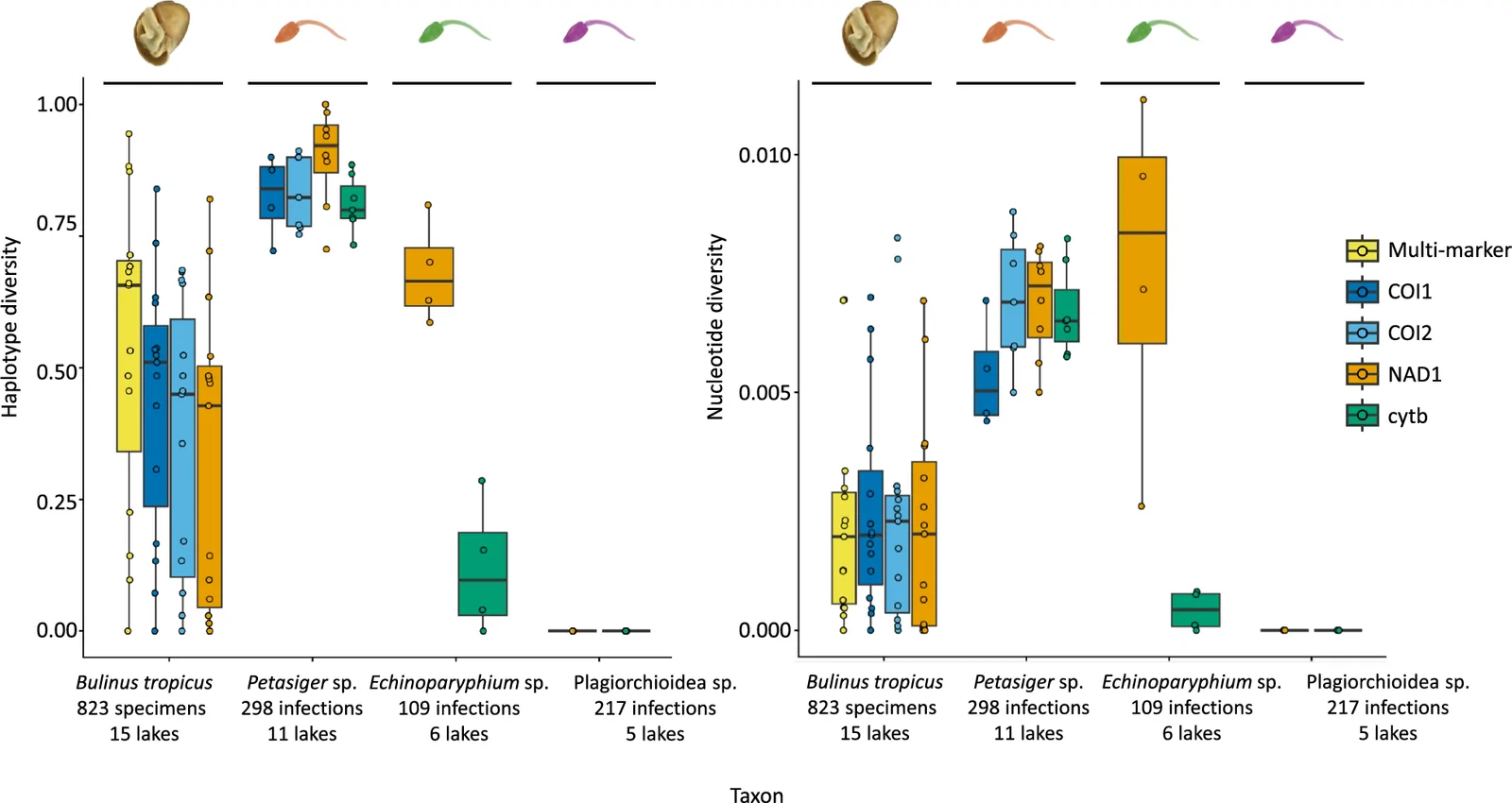
Variation in haplotype and nucleotide diversity in populations of *Bulinus tropicus*, *Petasiger *sp., *Echinoparyphium* sp. and Plagiorchioidea sp. depending on the genetic marker analysed, and only including populations represented by five or more specimens. Median diversity values are shown as thick horizontal lines within the boxes; the upper and lower box edges represent the first and third quartiles. Each dot represents a single population. *COI1* cytochrome c oxidase subunit 1 mitochondrial gene fragment 1, *COI2* cytochrome c oxidase subunit 1 mitochondrial gene fragment 2, *NAD1* NADH dehydrogenase subunit I gene, *cytb* cytochrome b gene. Colouring of cercariae pictograms for illustrative purposes only

The three sequenced *B. tropicus* markers (COI1, COI2, NAD1) revealed similar patterns of genetic diversity, with approximately 40 unique haplotypes observed in each dataset (Table S3). The mean number of unique haplotypes per lake was 4.2 [range 1–12] for COI1, 3.5 [1–11] for COI2, 3.9 [1–15] for NAD1 and 7.3 [1–25] in the multi-marker alignment. Pairwise Wilcoxon tests for *B. tropicus* do not indicate significant differences in median haplotype or nucleotide diversity for these three markers (Table S4, Fig. 2). The number of unique haplotypes in *Petasiger* sp. populations differs among markers, with the mean number of unique haplotypes per lake being 4.2 [1–10] for COI1, 8.2 [1–21] for COI2, 14.9 [1–39] for NAD1 and 6.0 [1–15] for cyb, but this variation also depends on sequencing success (COI1 was only successfully sequenced 71 times *versus* NAD1 298 times). Haplotype diversity in *Petasiger* sp. populations does not significantly differ among the COI1, COI2, NAD1 and cytb markers (Table S4; Fig. 2). Similarly, no differences were found in the nucleotide diversity of this species. For *Echinoparyphium* sp., the mean number of unique haplotypes per lake was 1.5 [1–2] for COI2, 4.0 [2–9] for NAD1 and 1.7 [1–2] for cyb. Furthermore, haplotype and nucleotide diversities were significantly higher in NAD1 compared to cytb for this species (Table S4). Finally, only one unique haplotype is observed in NAD1 and cytb of Plagiorchioidea sp., out of 217 and 210 sequences, respectively. These sequences come from five lakes in the Bunyaruguru cluster, and one lake, with only one infection, in the Ndali-Kasenda cluster (Table 1). Therefore, haplotype and nucleotide diversities were null for this species (Table S3, Fig. 2).

When comparing genetic diversity among species, within the same markers, pairwise Wilcoxon tests show that both haplotype and nucleotide diversities are higher in *Petasiger* sp. compared to *B. tropicus* in all markers (Table S5, Fig. 2). Comparison of genetic diversity between *B. tropicus* and *Echinoparyphium* sp. in NAD1 also reveals significantly higher median values of haplotype and nucleotide diversity in the parasite (Table S5, Fig. 2). Finally, comparing genetic diversity in NAD1 and cytb between *Petasiger* sp. and *Echinoparyphium* sp. shows that in the latter species both markers have significantly lower median values of haplotype diversity, but nucleotide diversity is only significantly lower in cytb of *Echinoparyphium* sp. (Table S5, Fig. 2).

The hypothesis of neutral evolution is not rejected in most of the 15 *B. tropicus* populations (Table S6), although differences exist among markers and tests. For example, significantly negative Tajima’s *D* values are obtained in all markers for the *B. tropicus* population of Lake Nyungu, whereas no significant deviation from neutrality is detected in any marker for the same population using Fu’s *Fs* (Table S6). In addition, Tajima’s *D* and Fu’s *Fs* indicate significant departure from neutrality more often in COI2 and NAD1 than in COI1. Deviation from neutrality is detected in six out of eight *Petasiger* sp. populations, based on large negative values for Fu’s *Fs* in NAD1. However, in five of these six populations this pattern was not confirmed by tests on Tajima’s *D* in NAD1, nor in four by tests on Fu’s *Fs* or Tajima’s D in other markers. Finally, neutrality is not rejected in any of the four retained populations of *Echinoparyphium* sp. (Table S6). Neutrality was not tested in Plagiorchioidea sp. populations due to their lack of genetic variability.

### Genetic structure of snail and trematode populations

**Fig. 3:**
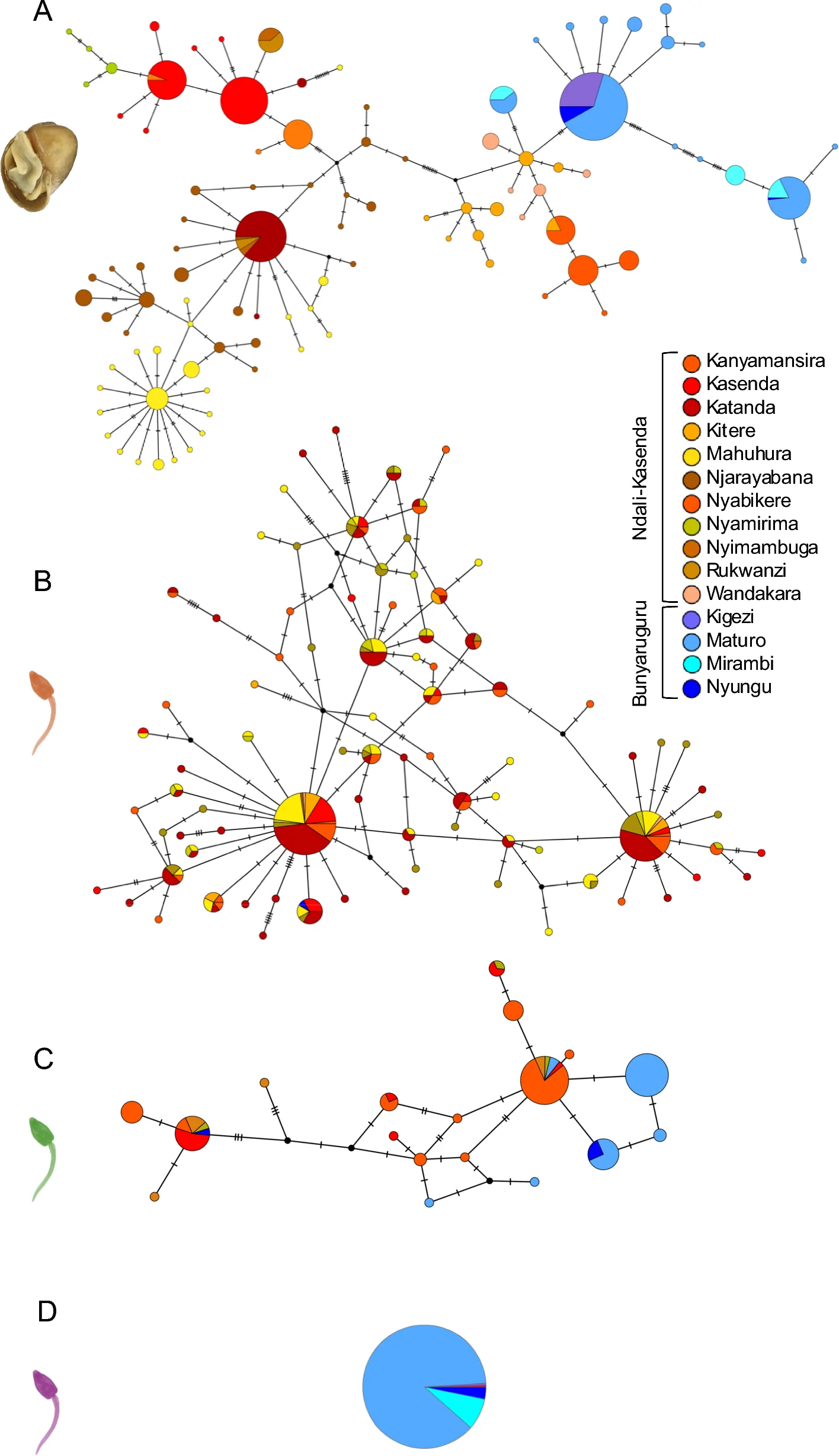
TCS haplotype networks based on the multi-marker dataset of *Bulinus tropicus* (**A**; *n* = 808) and the NADH dehydrogenase subunit I gene for parasite species: *Petasiger* sp. (**B**; *n* = 298), *Echinoparyphium* sp. (**C**; *n* = 109) and Plagiorchioidea sp. (**D**;* n* = 217). Each circle represents a unique haplotype, and circle size is proportional to the number of specimens carrying that haplotype. The smallest circles represent one specimen, whereas the largest circles in **A**, **B**, **C** and **D** represent respectively 165, 74 and 29 and 217 specimens. Coloring of pie parts within circles corresponds to populations, with lakes of the Ndali-Kasenda cluster indicated in shades of red to yellow, and those of the Bunyaruguru cluster in shades of blue. Dashes on segments connecting haplotype circles represent the number of inferred mutations between haplotypes. Colouring of cercariae pictograms for illustrative purposes only

**Fig. 4:**
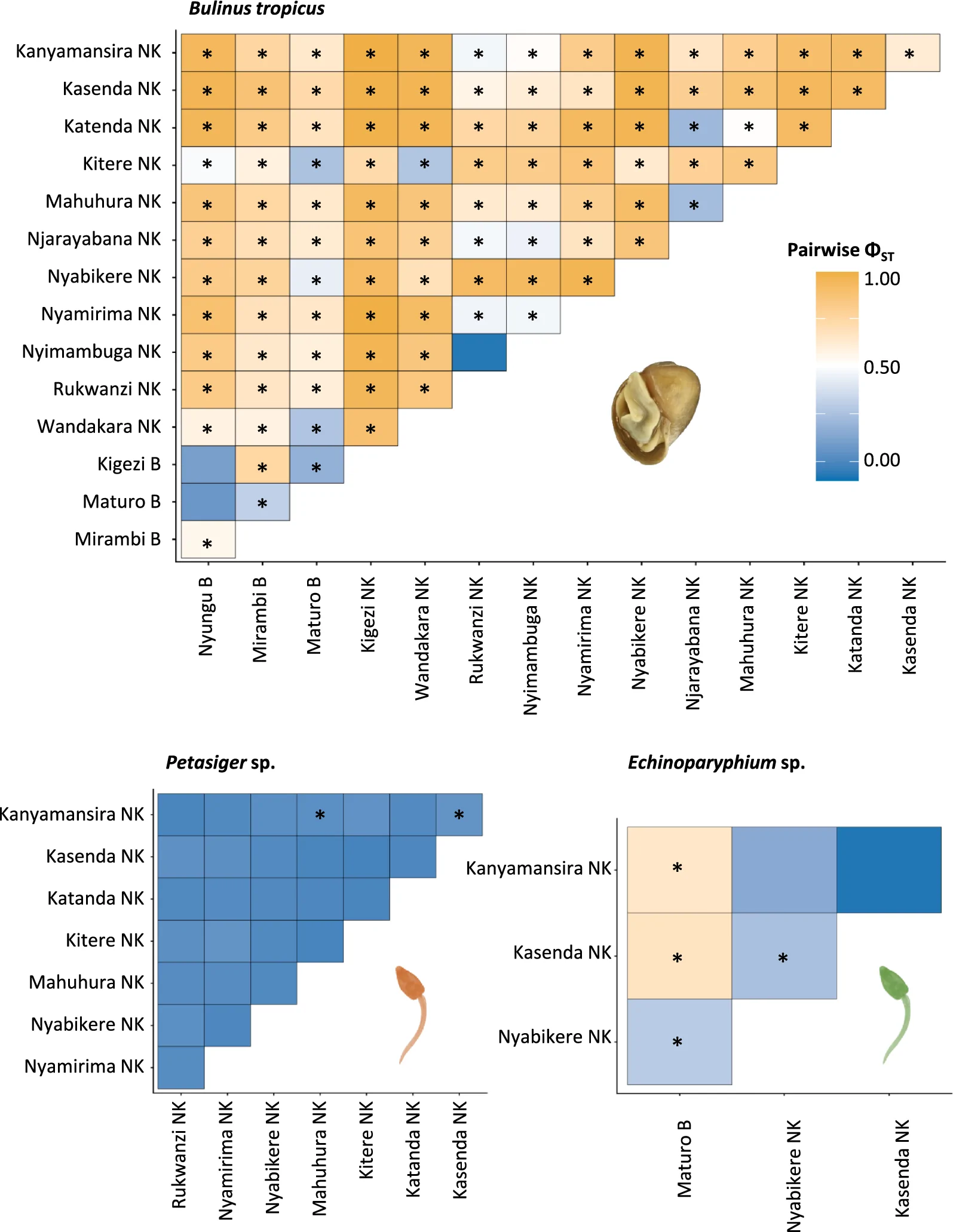
Heatmaps of the pairwise fixation index (pairwise Φ_ST_) among populations of *Bulinus tropicus* (based on the multi-marker dataset), *Petasiger* sp. and *Echinoparyphium* sp. (both based on the NADH dehydrogenase subunit I marker). The hypothesis that pairwise Φ_ST_ differs from zero was tested using analyses of molecular variance with 10,000 permutations. Significant pairwise differentiation (*P* < 0.05) is indicated with asterisks. The suffixes NK and B differentiate lakes of the Ndali-Kasenda and Bunyaruguru lake clusters

No single haplotype of *B. tropicus* is dominant at the scale of an entire crater lake cluster, but for individual lakes (i.e. populations) dominance of one or a few closely related haplotypes is observed in the multi-marker dataset and in all mitochondrial markers individually (Figs. 3, S1). We find significant differentiation among populations of *B. tropicus* across all markers, both between the Ndali-Kasenda and Bunyaruguru lake clusters and among lakes within each cluster (Table 2). Furthermore, > 90% of pairwise AMOVA tests performed on *B. tropicus* markers also indicate significant genetic differentiation between pairs of lakes (Fig. 4). For trematodes, differentiation was not tested at the lake cluster level because in the Bunyaruguru cluster only one *Petasiger* sp. specimen was found (Lake Nyungu), and sample size from only one population of *Echinoparyphium* sp. (Lake Maturo) is sufficiently large (≥ 5) for analysis. Furthermore, the lack of genetic variability among populations of Plagiorchioidea sp. precludes testing for genetic differentiation. For *Petasiger* sp., no differentiation among populations was detected using AMOVA, instead > 99% of the genetic variability is observed within populations (Table 2). In terms of pairwise comparisons among lakes, < 10% of the AMOVA tests are significant across all markers (Fig. 4). Finally, the AMOVA results for *Echinoparyphium* sp. differ between the markers analysed: general and pairwise AMOVAs indicate significant differentiation among populations for NAD1 (Table 2, Fig. 4), but not for cytb.

**Table 2:** Results of the analysis of molecular variance for each species-marker combination

Species	Marker	Source of variation	Df	Variation explained	Fixation indices	*P*
*Bulinus tropicus*	Multi-markers *n* = 808, 15 lakes	Between clusters	1	32.5%	Φ_CT_ = 0.32	< 0.050
		Among lakes within clusters	13	45.2%	Φ_SC_ = 0.67	< 0.001
		Within lakes	793	22.2%	Na	Na
		Non-hierarchical among lakes	Na	Na	Φ_ST_ = 0.78	< 0.001
	COI1 *n* = 823, 15 lakes	Between clusters	1	34.6%	Φ_CT_ = 0.35	< 0.050
		Among lakes within clusters	13	40.6%	Φ_SC_ = 0.62	< 0.001
		Within lakes	808	24.8%	Na	Na
		Non-hierarchical among lakes	Na	Na	Φ_ST_ = 0.75	< 0.001
	COI2 *n* = 812, 15 lakes	Between clusters	1	44.8%	Φ_CT_ = 0.45	< 0.001
		Among lakes within clusters	13	31.1%	Φ_SC_ = 0.56	< 0.001
		Within lakes	797	24.0%	Na	Na
		Non-hierarchical among lakes	Na	Na	Φ_ST_ = 0.76	< 0.001
	NAD1 *n* = 816, 15 lakes	Between clusters	1	24.7%	Φ_CT_ = 0.25	< 0.050
		Among lakes within clusters	13	55.1%	Φ_SC_ = 0.73	< 0.001
		Within lakes	801	20.2%	Na	Na
		Non-hierarchical among lakes	Na	Na	Φ_ST_ = 0.80	< 0.001
*Petasiger* sp.	COI1 *n* = 59, 4 lakes	Among lakes	3	1.2%	Φ_ST_ = 0.01	> 0.100
		Within lakes	56	98.8%		
	COI2 *n* = 168, 7 lakes	Among lakes	6	1.3%	Φ_ST_ = 0.01	> 0.100
		Within lakes	162	98.7%		
	cytb *n* = 125, 7 lakes	Among lakes	6	0.0%	Φ_ST_ = 0.01	> 0.100
		Within lakes	119	100.0%		
	NAD1 *n* = 293, 8 lakes	Among lakes	7	1.0%	Φ_ST_ = 0.01	> 0.050
		Within lakes	286	99.0%		
*Echinoparyphium* sp.	cytb *n* = 108, 4 lakes	Among lakes	3	6.6%	Φ_ST_ = 0.07	> 0.050
		Within lakes	105	93.4%		
	NAD1 *n* = 101, 4 lakes	Among lakes	3	35.3%	Φ_ST_ = 0.35	< 0.001
		Within lakes	98	64.7%		

*Φ*_*CT*_ differentiation among lake clusters, *Φ*_*SC*_ differentiation among lakes within the same cluster, *Φ*_*ST*_ non-hierarchical differentiation among lakes

*Df* degree(s) of freedom, *Na* not applicable, *COI1* cytochrome c oxidase subunit 1 mitochondrial gene fragment 1, *NAD1* NADH dehydrogenase subunit I gene, *cytb* cytochrome b gene

Mantel tests yielded evidence for isolation-by-distance among the *B. tropicus* populations as Slatkin’s genetic distances correlate significantly with geographic distances among lakes using the COI1 and COI2 datasets (*P* < 0.05, Table S7), but the signal was non-significant for NAD1 (*P* > 0.1) and only approached significance using the multi-marker alignment (*P* = 0.052). For both parasite species, Slatkin’s genetic distances among populations do not correlate significantly with geographic distance (Table S7, Fig. S2). However, for *Echinoparyphium* sp., the Slatkin’s distances among lakes from the same cluster appear lower than those among lakes from different clusters, which suggests that some dispersal limitation exists at the scale of ~ 100 km, although our current sample size at this scale is insufficient for formal testing (Table S7, Fig. S2).

## Discussion

We aimed at investigating how complex life cycles involving multiple host organisms affect the dispersal capacity of parasitic trematodes. The crater lakes of western Uganda provide an exceptional study system to address such questions because they encompass a large number of hydrographically isolated lake catchments. These lakes are separated by distances ranging from a few hundred meters between certain lakes within a lake cluster (e.g., Nyungu and neighbouring Maturo in the Bunyaruguru cluster: Fig. 1) to ~ 100 km between lakes of different lake clusters. Whereas lake catchment boundaries represent strong barriers to dispersal for aquatic snails which serve as intermediate host, final host organisms mediate parasite dispersal over larger distances. From a practical and ethical viewpoint it is more feasible to sample the trematode stages from the snail host than from vertebrates, even though the cercarial population structure and diversity may not fully reflect patterns in the adult population (e.g., [58]). Here we zoomed in on three different trematode species and show how the inferred population genetic structure can strongly differ and inform on processes occurring at other stages of the life cycle, such as dispersal.

Our results indicate contrasting levels of gene flow, genetic diversity and population structure among the three examined parasite species and their intermediate host snail. Haplotype and nucleotide diversity were twice as high in populations of *Petasiger* sp. compared to the intermediate host *B. tropicus,* and unlike their snail hosts, AMOVA tests indicated an absence of population differentiation in the parasite. Strikingly, comparison with specimens collected in Kenya by [35] and in South Africa by [22] reveals the presence of shared NAD1 haplotypes in all countries (distance 400–3,000 km; data not shown). Those patterns corroborate our hypothesis that the final host facilitates gene flow for *Petasiger* sp., even at a large geographic scale [5, 6, 59], creating a single (‘panmictic’) region-wide population. Indeed, *Petasiger* sp. presumably infects piscivorous birds such as reed cormorants that regularly disperse over distances of 10–100 km [60, 61], which would therewith prevent parasite population differentiation via founder effects or genetic drift at the geographic scale studied here. The occurrence of shared haplotypes across Uganda, Kenya and South Africa also fits with the capacity of the reed cormorant to occasionally disperse over even larger distances to find new breeding or foraging grounds [61]. Consequently, the dispersal of *Petasiger* sp. likely does not depend on the dispersal capacities of the snail intermediate host or of the amphibians or fish serving as second intermediate hosts. Exchanges with remote populations and large effective population size may be at the origin of the high genetic diversity observed in the crater lake populations of *Petasiger* sp., increasing the adaptative potential of this species [62].

Contrastingly, the significant genetic differentiation among populations of *Echinoparyphium* sp. suggests that its most mobile host disperses at the scale of lakes and lake clusters, thus less than the final host of *Petasiger* sp. but considerably more than the intermediate host snail. Information on the life cycle of *Echinoparyphium* is incomplete but it likely infects a similar range of final hosts as *E. elegans*, the only described *Echinoparyphium* species transmitted by *B. tropicus* [39]. The only known host animal of *E. elegans* documented by field observations is the cattle egret (*Bubulcus ibis*), but experimental infections show that the parasite can also complete its life cycle in small mammals and domesticated birds [39], and therefore, the final hosts of *Echinoparyphium* sp. in our study region are most likely birds and small mammals. These hosts would imply that the parasite is allogenic with substantial dispersal among neighbouring lakes, but not among lake clusters.

Beyond host mobility, several other factors that were not investigated in this study may influence genetic differentiation in parasites. For example, high host specificity, low effective population size and self-fertilisation are linked to high differentiation among populations [62]. Our results revealed some differences among markers for *Echinoparyphium* sp., perhaps reflecting different selective constraints acting on these markers. In this species, diversity in NAD1 was significantly higher than in cytb, resulting in significant genetic structuring among populations for the first but not the second marker. Those differences may indicate that cytb may be under stabilizing selection, but neither Tajima’s *D* nor Fu’s *Fs* tests indicate deviation from neutrality.

Finally, we observed a single haplotype without genetic variation in NAD1 and cytb of Plagiorchioidea sp., for which the life cycle remains unknown. This species was collected in five neighbouring lakes, and we suspect that the observed lack of genetic diversity results from a recent introduction to the crater lake area of a very small founding population (possibly one or a few adults infecting one final host). This colonization likely started in Lake Maturo, where most infections are concentrated. Newly established populations of parasites with complex life cycles may be more genetically depauperate than new populations of free-living organisms due to the possibly smaller founding population size and lower probability of successful colonization [63, 64]. Furthermore, autogenic trematode species may undergo stronger founder effects than allogenic species, at least in clusters of hydrographically isolated lakes, because colonisation and subsequent migration are potentially more restricted [30, 64]. Despite the limited genetic diversity, Plagiorchioidea sp. has been highly successful in establishing in Lake Maturo, as it infects > 90% of the ~ 200 snails tested [13]. Although its final host is unknown, Plagiorchioidea sp. may be autogenic, in which case a plausible explanation for its patterns of genetic diversity is anthropogenic dispersal via fishes used to restock Lake Maturo and, secondarily, surrounding crater lakes, after which Plagiorchioidea sp. may have found a highly competent host in *B. tropicus*. It would be interesting to compare autogenic and allogenic species in contexts where long-distance aquatic dispersal is possible, as dispersal dynamics of the aquatic intermediate and final hosts may synergistically enhance trematode dispersal in such systems, but not in the crater lakes studied here.

The genetic diversity observed for *B. tropicus* in all sequenced markers is comparable to values obtained for other molluscs using similar markers: mean haplotype diversity in COI1 across all populations was 0.44 in our study (ranging from 0.00 to 0.84), compared to 0.67 for the same marker across populations of the hygrophilid *Indoplanorbis exustus* from multiple south-eastern Asian countries [65] or 0.33–0.78 in populations of *B. truncatus* from various African countries [66]. Our results are also in line with the high number of unique COI haplotypes observed in *B. tropicus* populations from the Ugandan crater lakes by [14]. Given the relatively recent formation of the crater lakes (8,000–50,000 years ago), [14] concluded that the substantial genetic diversity observed in *B. tropicus* unlikely results from genetic differentiation after colonisation by a single population. Although the crater lakes are largely hydrographically isolated (some crater lakes in the Bunyaruguru cluster are inter-connected via streams), there are several larger water bodies in western Uganda (the Nile River and lakes Victoria, Edward, George and Albert) that may have served as sources for multiple independent colonisation events from various regional sources [14], as appears to be the case for cichlid fishes [67]. Consequently the founding populations in various lakes may have had different genetic compositions. Beyond multiple colonisation events, our results further suggest that neighbouring lakes shared small numbers of inter-lake effective migrants, which explains the observed pattern of isolation-by-distance. The co-occurrence of genetically distant haplotypes within the same lake corroborates both independent colonisations and low-frequency short-distance dispersal.

Additionally, genetic drift fostered by limited inter-lake gene flow due to restricted dispersal probably also contributed to the genetic characteristics of these host snail populations in the crater lakes, as observed in other populations of *B. tropicus* and other *Bulinus* species [28, 68–70]. Indeed, our *B. tropicus* populations are significantly genetically differentiated from one another among both crater lakes and lake clusters, in all markers, even between lakes separated by a few kilometres only. Therefore, even at the km scale, inter-lake gene flow is limited, which is corroborated by very high pairwise Φ_ST_ values (0.71 on average). Although Φ_ST_ values estimated from mitochondrial markers are challenging to interpret (given the haploid, non-recombining nature and maternal inheritance), similar patterns and high pairwise F_st_ values are also observed among populations of cichlids in neighbouring crater lakes [67] and among populations of *B. truncatus* both at continental and regional scales [28] from nuclear data. Second, genetic differentiation between lakes tends to be positively correlated with geographical distance, although in our data this correlation is only significant for COI1 and COI2, not for NAD1 despite similar sample sizes and genetic diversity in each marker. Isolation by dispersal limitation (sensu [54]) at similar geographical scale in *B. truncatus* [70] and *B. globosus* [71] indicates that dispersal in these host snails most commonly happens over short distances. A pattern of frequent short-range dispersal alongside rare long-range dispersal (so-called stratified dispersal) has also been described in other snail species [72]. In our study system, dispersal of snails by means of adults or eggs attached to fishing nets which are used in several lakes may also contribute to the low levels of gene flow among neighbouring lakes. Our findings on genetic differentiation among populations of *B. tropicus* in the Ugandan crater lakes resemble data on cichlid fishes inhabiting these lakes, namely strong genetic differentiation among lakes and lake clusters [67], and between Lake Saka and the adjacent Mpanga River in the Fort Portal cluster [73].

## Conclusions

In this study, we compared the genetic diversity and population structure of three snail-borne parasite species and their first intermediate host snail across a system of hydrographically isolated freshwater lakes in rural Africa. We then interpreted these genetic patterns in light of differences in the ecology and life-history traits of the parasites to infer dispersal and connectivity. The three parasite species and their snail host exhibited highly contrasting patterns of genetic diversity and population differentiation. The parasite species with the most mobile final host (*Petasiger* sp.), however, showed the highest genetic diversity, likely due to frequent mixing of parasite populations (panmixia), which results in large effective population size and limited genetic differentiation. Our data suggest intermediate host mobility for *Echinoparyphium* sp. given the moderate level of population structuring. Finally, the complete lack of genetic variation in Plagiorchioidea sp. suggests a recent genetic bottleneck probably related to recent establishment in the crater lakes with limited genetic diversity in the founding population. The high genetic diversity and strong population structure of the intermediate host snail *B. tropicus* imply that they may have established in the area through multiple independent colonisation events from various regional sources, with subsequent low inter-lake effective migration rates, and substantial genetic drift in small populations. This high genetic diversity suggests that these populations could be able to locally adapt to new selective challenges imposed by trematode infections. Despite the important knowledge gaps on the ecology and diversity of African trematodes, our results show that genetic data can be leveraged to gain insights into the life cycles and dispersal capabilities of understudied parasite populations.

## Supplementary Information

Supplementary material 1: Table S1: Infection prevalence (in %) of the three parasites in the *Bulinus tropicus* populations analysed. Sample sizes (*n*) represent the number of host snails screened for infection per lake. Table S2: GenBank accession numbers of the sequences used in the study. Table S3: Sample size (*n*), number of unique haplotypes (Nh), number of polymorphic sites (Np), haplotype diversity (h) and nucleotide diversity (π) with standard deviation in the various populations of *Bulinus tropicus*, *Petasiger* sp., *Echinoparyphium* sp. and Plagiorchioidea sp. Table S4: *P*-values obtained from the pairwise Wilcoxon tests with Hommel’s corrections to compare haplotype and nucleotide diversity between analysed markers for each species. Populations of Plagiorchioidea sp. were not included as no genetic polymorphism was observed within or among these populations. Populations with sample size < 5 are excluded from these analyses. Values in bold indicate significant differences. Cells containing – indicate that no comparison was performed because the markers could not be amplified. Table S5: *P*-values obtained from the pairwise Wilcoxon tests with Hommel’s corrections to compare haplotype and nucleotide diversity between the species analysed for each marker sequenced. Populations of Plagiorchioidea sp. were not included as no genetic polymorphism was observed within or among these populations. Populations with sample size < 5 are excluded from these analyses. Values in bold indicate significant differences. Table S6: Tajima’s D and Fu’s Fs tests of neutral evolution. Lakes with low sample size (< 5 are excluded) and lakes with only 1 haplotype are indicated with –. Significance of p-value is signalled by * if < 0.05 for Tajima’s D or < 0.02 for Fu’s Fs and ** if < 0.001 (both tests). Table S7: Results of Mantel tests to examine correlations between Slatkin’s genetic distance among populations and the Euclidian geographic distance between the lakes where these populations were sampled. Figure S1: TCS haplotype networks of: A. COI1 marker of *Bulinus tropicus* (*n* = 823); B. COI2 marker of *B. tropicus* (*n* = 812); C. NAD1 marker of *B. tropicus* (*n *= 816); D. COI1 of *Petasiger* sp. (*n* = 71); ); E. COI2 of *Petasiger* sp. (*n* = 171); ); F. cytb of *Petasiger* sp. (*n* = 128); G. cytb of *Echinoparyphium* sp. (*n* = 116). Each circle represents a unique haplotype, circle size being proportional to the number of specimens with the haplotype/ Colors proportions in the circles provides information on the population of the specimens for each population (blue colors = populations from the Bunyaruguru cluster, red to yellow colors = populations from the Ndali-Kasenda cluster). Dashes on segments connecting haplotype circles represent the number of inferred mutation steps between haplotypes. Figure S2 Relationships between Slatkin’s genetic distance among populations and the geographic distance between the lakes where the populations were sampled for *Bulinus tropicus* (multi-locus dataset, 15 lakes), *Petasiger* sp. (NAD1 marker, eight lakes) and *Echinoparyphium* sp. (NAD1 marker, four lakes). A linear regression line is fitted for illustration in the plot of B. tropicus as a near-significant isolation-by-distance pattern was observed using a Mantel test (*P* = 0.052). Geographic distances were log10-transformed in the *B. tropicus* plot to increase visual clarity.

## Data Availability

Dried DNA vouchers are stored at the Royal Museum for Central Africa (Tervuren, Belgium) under BE.RMCA.MOL.DNA reference numbers 1–2472. Raw Illumina sequencing read sequences are stored in the Sequence Read Archive accession with the identifier PRJNA1227448. Quality-controlled DNA sequences of trematode parasites are archived on GenBank with accession numbers OQ541006–OQ541828; OQ541851–OQ542662; OQ558014–OQ558829; OQ543471–OQ543551; Q606526–OQ606696; OQ574003–OQ574300; OQ574934–OQ575061; OQ606482–OQ60649; OQ573822–OQ573932; OQ574693–OQ574808; OQ574304–OQ574520; OQ575062–OQ575271.

## Abbreviations

HTAS: High-throughput amplicon sequencing
BLAST: Basic Local Alignment Search Tool
RD-PCR: Rapid-diagnostic multiplex polymerase chain reaction RD-PCR
COI1: Cytochrome c oxidase subunit 1 mitochondrial gene fragment 1
NAD1: NADH dehydrogenase subunit I gene
ITS1: Internal transcribed spacer 1
cytb: Cytochrome b
ASV: Amplicon sequence variant
NUMT: Nuclear mitochondrial DNA sequences
AMOVA: Analysis of molecular variance
TCS: Templeton, Crandall and Sing
Df: Degrees of freedom
Na: Not applicable
