# Supplementary material for: Contrasting patterns of trematode and host snail dispersal across clusters of tropical crater lakes
Source: Infect Dis Poverty. 2026 Aug 24;15:92. doi: 10.1186/s40249-026-01488-9 (PMC13501632; doi:10.1186/s40249-026-01488-9)
Supplement: Supplementary file 1 [file 40249_2026_1488_MOESM1_ESM.docx]

**Supplementary materials**

**Contrasting patterns of trematode and host snail dispersal across clusters of tropical crater lakes**

Cyril Hammoud^1,2^, Bert Van Bocxlaer^3,1^, Julius Tumusiime^4^, Dirk Verschuren^1^, Christian Albrecht^4,5^, Casim Umba Tolo^4^, Tine Huyse^2,6^

1. Ghent University, Limnology Unit, Department of Biology, 9000 Gent, Belgium

2. Royal Museum for Central Africa, Department of Biology, 3080 Tervuren, Belgium

3. French National Centre for Scientific Research, University of Lille, Mixed research unit 8198 Evolution-Ecology-Paleontology, F-59000 Lille, France

4. Mbarara University of Science and Technology, Department of Biology, 40006 Mbarara, Uganda

5. Justus Liebig University, Systematics and Biodiversity Laboratory, Department of Animal Ecology and Systematics, D-35392 Giessen, Germany

6. University of Leuven, Laboratory of Biodiversity and Evolutionary Genomics, B-3000 Leuven, Belgium

Corresponding author: Cyril Hammoud (cyril.hammoud@nioz.nl)

**Clinical trial number**: Not applicable.

|  | | Latitude | Longitude | *Petasiger* sp. | *Echinoparyphium* sp. | Plagiorchioidea sp. |
| --- | --- | --- | --- | --- | --- | --- |
| Bunyaruguru | Nyungu n=115 | -0.25772 | 30.0943 | 0.9 | 4.3 | 7 |
|  | Maturo n=208 | -0.26555 | 30.10468 | 0 | 26.9 | 91.3 |
|  | Kigezi n=85 | -0.28746 | 30.109867 | 0 | 0 | 2.4 |
|  | Mirambi n=87 | -0.22515 | 30.10787 | 0 | 0 | 20.7 |
| Ndali-Kasenda | Nyabikere n=200 | 0.49675 | 30.3311 | 1.5 | 22 | 0 |
|  | Kitere n=200 | 0.3973 | 30.2734 | 6.5 | 0 | 0 |
|  | Wandakara n=89 | 0.41565 | 30.2705 | 2.2 | 0 | 0 |
|  | Kasenda n=419 | 0.433017 | 30.29143 | 5 | 3.3 | 0.2 |
|  | Njarayabana n=73 | 0.429017 | 30.25103 | 1.4 | 0 | 0 |
|  | Mahuhura n=200 | 0.43925 | 30.26577 | 16.5 | 0 | 0 |
|  | Nyamirima n=111 | 0.519967 | 30.32038 | 4.5 | 2.7 | 0 |
|  | Rukwanzi n=201 | 0.473983 | 30.27928 | 8.5 | 0 | 0 |
|  | Nyinambuga n=71 | 0.481133 | 30.28778 | 0 | 0 | 0 |
|  | Katanda n=200 | 0.47795 | 30.26145 | 31.5 | 0 | 0 |
|  | Kanyamansira n=57 | 0.414683 | 30.3046 | 35.1 | 14 | 0 |

*Table S1: Infection prevalence (in %) of the three parasites in the* Bulinus tropicus *populations analysed. Sample sizes (n) represent the number of host snails screened for infection per lake.*

*Table S2: GenBank accession numbers of the sequences used in the study.*

| Organism | Marker | GenBank accession numbers |
| --- | --- | --- |
| *Bulinus tropicus* | cox1 I | OQ541006-OQ541828 |
|  | cox1 II | OQ541851-OQ542662 |
|  | NAD1 | OQ558014-OQ558829 |
| *Petasiger* sp. | cox1 I | OQ543471-OQ543551 |
|  | cox1 II | OQ606526- OQ606696 |
|  | NAD1 | OQ574003-OQ574300 |
|  | cytb | OQ574934-OQ575061 |
| *Echinoparyphium* sp. | cox1 I | - |
|  | cox1 II | OQ606482- OQ606490 |
|  | NAD1 | OQ573822-OQ573932 |
|  | cytb | OQ574693-OQ574808 |
| Plagiorchioidea sp. | cox1 I | - |
|  | cox1 II | - |
|  | NAD1 | OQ574304-OQ574520 |
|  | cytb | OQ575062-OQ575271 |

*Table S3: Sample size (n), number of unique haplotypes (Nh), number of polymorphic sites (Np), haplotype diversity (h) and nucleotide diversity (π) with standard deviation in the various populations of* Bulinus tropicus*,* Petasiger *sp.,* Echinoparyphium *sp. and Plagiorchioidea sp.*

| Taxon | Marker | Cluster | Lake | n | Nh | Np | h | π |
| --- | --- | --- | --- | --- | --- | --- | --- | --- |
| *Bulinus tropicus* | Multi-markers | Bunyaruguru | Nyungu | 14 | 2 | 20 | 0.1429 ± 0.1188 | 0.002199 ± 0.001382 |
|  |  |  | Maturo | 191 | 14 | 32 | 0.6569 ± 0.0282 | 0.006949 ± 0.003556 |
|  |  |  | Kigezi | 47 | 1 | 0 | 0.0000 ± 0.0000 | 0.000000 ± 0.000000 |
|  |  |  | Mirambi | 34 | 3 | 21 | 0.6827 ± 0.0236 | 0.006934 ± 0.003640 |
|  |  | Ndali-Kasenda | Nyabikere | 44 | 5 | 4 | 0.6607 ± 0.0287 | 0.000631 ± 0.000508 |
|  |  |  | Kitere | 33 | 9 | 13 | 0.8826 ± 0.0238 | 0.002312 ± 0.001377 |
|  |  |  | Wandakara | 18 | 5 | 6 | 0.6928 ± 0.0864 | 0.001263 ± 0.000873 |
|  |  |  | Kasenda | 133 | 7 | 8 | 0.5324 ± 0.0260 | 0.000478 ± 0.000419 |
|  |  |  | Njarayabana | 64 | 25 | 28 | 0.9444 ± 0.0127 | 0.003349 ± 0.001860 |
|  |  |  | Mahuhura | 54 | 24 | 34 | 0.8728 ± 0.0354 | 0.001968 ± 0.001191 |
|  |  |  | Nyamirima | 7 | 4 | 5 | 0.7143 ± 0.1809 | 0.001246 ± 0.000955 |
|  |  |  | Rukwanzi | 19 | 2 | 8 | 0.4561 ± 0.0852 | 0.002809 ± 0.001663 |
|  |  |  | Nyinambuga | 12 | 2 | 8 | 0.4848 ± 0.1059 | 0.002986 ± 0.001814 |
|  |  |  | Katanda | 80 | 3 | 9 | 0.0972 ± 0.0448 | 0.000469 ± 0.000417 |
|  |  |  | Kanyamansira | 33 | 3 | 3 | 0.2254 ± 0.0916 | 0.000309 ± 0.000327 |
|  | COI1 | Bunyaruguru | Nyungu | 15 | 2 | 7 | 0.1333 ± 0.1123 | 0.001973 ± 0.001609 |
|  |  |  | Maturo | 191 | 8 | 13 | 0.6233 ± 0.0283 | 0.006907 ± 0.003954 |
|  |  |  | Kigezi | 47 | 1 | 0 | 0.0000 ± 0.0000 | 0.000000 ± 0.000000 |
|  |  |  | Mirambi | 34 | 2 | 7 | 0.4278 ± 0.0667 | 0.006331 ± 0.003769 |
|  |  | Ndali-Kasenda | Nyabikere | 69 | 3 | 2 | 0.6338 ± 0.0283 | 0.001610 ± 0.001327 |
|  |  |  | Kitere | 33 | 5 | 5 | 0.7367 ± 0.0400 | 0.002867 ± 0.002023 |
|  |  |  | Wandakara | 18 | 3 | 2 | 0.3072 ± 0.1316 | 0.000677 ± 0.000805 |
|  |  |  | Kasenda | 141 | 6 | 5 | 0.5370 ± 0.0227 | 0.001245 ± 0.001113 |
|  |  |  | Njarayabana | 64 | 12 | 13 | 0.8398 ± 0.0245 | 0.005696 ± 0.003404 |
|  |  |  | Mahuhura | 57 | 9 | 10 | 0.5345 ± 0.0729 | 0.003828 ± 0.002484 |
|  |  |  | Nyamirima | 7 | 3 | 3 | 0.5238 ± 0.2086 | 0.001812 ± 0.001652 |
|  |  |  | Rukwanzi | 20 | 3 | 3 | 0.5105 ± 0.0907 | 0.002237 ± 0.001726 |
|  |  |  | Nyinambuga | 12 | 2 | 2 | 0.4848 ± 0.1059 | 0.002050 ± 0.001683 |
|  |  |  | Katanda | 81 | 2 | 3 | 0.0722 ± 0.0389 | 0.000458 ± 0.000615 |
|  |  |  | Kanyamansira | 34 | 2 | 1 | 0.1658 ± 0.0804 | 0.000350 ± 0.000539 |
|  | COI2 | Bunyaruguru | Nyungu | 15 | 2 | 6 | 0.1333 ± 0.1123 | 0.002410 ± 0.002063 |
|  |  |  | Maturo | 191 | 6 | 10 | 0.5238 ± 0.0306 | 0.008251 ± 0.004864 |
|  |  |  | Kigezi | 47 | 1 | 0 | 0.0000 ± 0.0000 | 0.000000 ± 0.000000 |
|  |  |  | Mirambi | 34 | 3 | 6 | 0.6827 ± 0.0236 | 0.007801 ± 0.004763 |
|  |  | Ndali-Kasenda | Nyabikere | 69 | 2 | 1 | 0.0290 ± 0.0279 | 0.000087 ± 0.000303 |
|  |  |  | Kitere | 33 | 3 | 2 | 0.3561 ± 0.0907 | 0.001107 ± 0.001213 |
|  |  |  | Wandakara | 18 | 4 | 3 | 0.6601 ± 0.0781 | 0.002559 ± 0.002124 |
|  |  |  | Kasenda | 135 | 3 | 2 | 0.0295 ± 0.0203 | 0.000089 ± 0.000305 |
|  |  |  | Njarayabana | 64 | 7 | 6 | 0.6855 ± 0.0463 | 0.003030 ± 0.002290 |
|  |  |  | Mahuhura | 54 | 11 | 10 | 0.4500 ± 0.0842 | 0.001718 ± 0.001572 |
|  |  |  | Nyamirima | 7 | 3 | 2 | 0.6667 ± 0.1598 | 0.002295 ± 0.002172 |
|  |  |  | Rukwanzi | 19 | 2 | 2 | 0.4561 ± 0.0852 | 0.002748 ± 0.002224 |
|  |  |  | Nyinambuga | 12 | 2 | 2 | 0.4848 ± 0.1059 | 0.002921 ± 0.002398 |
|  |  |  | Katanda | 81 | 2 | 1 | 0.0722 ± 0.0389 | 0.000218 ± 0.000484 |
|  |  |  | Kanyamansira | 33 | 2 | 1 | 0.1705 ± 0.0822 | 0.000513 ± 0.000781 |
|  | NAD1 | Bunyaruguru | Nyungu | 14 | 2 | 7 | 0.1429 ± 0.1188 | 0.002024 ± 0.001625 |
|  |  |  | Maturo | 191 | 4 | 9 | 0.5218 ± 0.0290 | 0.006114 ± 0.003545 |
|  |  |  | Kigezi | 47 | 1 | 0 | 0.0000 ± 0.0000 | 0.000000 ± 0.000000 |
|  |  |  | Mirambi | 34 | 2 | 8 | 0.4278 ± 0.0667 | 0.006928 ± 0.004037 |
|  |  | Ndali-Kasenda | Nyabikere | 69 | 2 | 1 | 0.0290 ± 0.0279 | 0.000059 ± 0.000204 |
|  |  |  | Kitere | 33 | 6 | 6 | 0.7216 ± 0.0498 | 0.002592 ± 0.001857 |
|  |  |  | Wandakara | 18 | 2 | 1 | 0.4706 ± 0.0823 | 0.000953 ± 0.000973 |
|  |  |  | Kasenda | 137 | 2 | 1 | 0.0146 ± 0.0143 | 0.000030 ± 0.000143 |
|  |  |  | Njarayabana | 64 | 12 | 12 | 0.8204 ± 0.0370 | 0.003201 ± 0.002140 |
|  |  |  | Mahuhura | 57 | 15 | 19 | 0.6347 ± 0.0716 | 0.002209 ± 0.001634 |
|  |  |  | Nyamirima | 7 | 1 | 0 | 0.0000 ± 0.0000 | 0.000000 ± 0.000000 |
|  |  |  | Rukwanzi | 20 | 2 | 4 | 0.4789 ± 0.0720 | 0.003878 ± 0.002572 |
|  |  |  | Nyinambuga | 12 | 2 | 4 | 0.4848 ± 0.1059 | 0.003926 ± 0.002694 |
|  |  |  | Katanda | 80 | 3 | 5 | 0.0972 ± 0.0448 | 0.000643 ± 0.000734 |
|  |  |  | Kanyamansira | 33 | 2 | 1 | 0.0606 ± 0.0561 | 0.000123 ± 0.000303 |
| *Petasiger* sp. | COI1 | Ndali-Kasenda | Nyabikere | 1 | 1 | 0 | 0.0000 ± 0.0000 | 0.000000 ± 0.000000 |
|  |  |  | Kitere | 5 | 4 | 6 | 0.9000 ± 0.1610 | 0.006931 ± 0.005118 |
|  |  |  | Wandakara | 1 | 1 | 0 | 0.0000 ± 0.0000 | 0.000000 ± 0.000000 |
|  |  |  | Kasenda | 18 | 9 | 9 | 0.8758 ± 0.0515 | 0.005501 ± 0.003557 |
|  |  |  | Mahuhura | 4 | 3 | 5 | 0.8333 ± 0.2224 | 0.006188 ± 0.004982 |
|  |  |  | Nyamirima | 2 | 2 | 1 | 1.0000 ± 0.5000 | 0.002475 ± 0.003501 |
|  |  |  | Rukwanzi | 9 | 5 | 8 | 0.7222 ± 0.1592 | 0.004400 ± 0.003178 |
|  |  |  | Katanda | 28 | 10 | 12 | 0.8042 ± 0.0590 | 0.004564 ± 0.003006 |
|  |  |  | Kanyamansira | 3 | 3 | 4 | 1.0000 ± 0.2722 | 0.006601 ± 0.005924 |
|  | COI2 | Ndali-Kasenda | Nyabikere | 1 | 1 | 0 | 0.0000 ± 0.0000 | 0.000000 ± 0.000000 |
|  |  |  | Kitere | 14 | 9 | 21 | 0.9121 ± 0.0593 | 0.005 ± 0.0593 |
|  |  |  | Wandakara | 1 | 1 | 0 | 0.0000 ± 0.0000 | 0.000000 ± 0.000000 |
|  |  |  | Kasenda | 20 | 12 | 14 | 0.9000 ± 0.0532 | 0.006899 ± 0.004385 |
|  |  |  | Mahuhura | 29 | 10 | 9 | 0.7660 ± 0.0613 | 0.005937 ± 0.003826 |
|  |  |  | Nyamirima | 5 | 4 | 6 | 0.9000 ± 0.1610 | 0.008798 ± 0.006425 |
|  |  |  | Rukwanzi | 15 | 7 | 7 | 0.7714 ± 0.1001 | 0.005977 ± 0.003983 |
|  |  |  | Katanda | 65 | 21 | 26 | 0.7534 ± 0.0467 | 0.008304 ± 0.004918 |
|  |  |  | Kanyamansira | 21 | 9 | 13 | 0.8238 ± 0.0600 | 0.007708 ± 0.004786 |
|  | NAD1 | Bunyaruguru | Nyungu | 1 | 1 | 0 | 0.0000 ± 0.0000 | 0.000000 ± 0.000000 |
|  |  | Ndali-Kasenda | Nyabikere | 6 | 6 | 9 | 1.0000 ± 0.0962 | 0.007660 ± 0.005212 |
|  |  |  | Kitere | 14 | 5 | 11 | 0.7253 ± 0.1040 | 0.005004 ± 0.003257 |
|  |  |  | Wandakara | 2 | 2 | 3 | 1.0000 ± 0.5000 | 0.006383 ± 0.007370 |
|  |  |  | Kasenda | 26 | 11 | 17 | 0.8062 ± 0.0706 | 0.005617 ± 0.003451 |
|  |  |  | Njarayabana | 1 | 1 | 0 | 0.0000 ± 0.0000 | 0.000000 ± 0.000000 |
|  |  |  | Mahuhura | 53 | 25 | 28 | 0.9035 ± 0.0312 | 0.006330 ± 0.003731 |
|  |  |  | Nyamirima | 17 | 15 | 13 | 0.9853 ± 0.0252 | 0.007541 ± 0.004513 |
|  |  |  | Rukwanzi | 33 | 23 | 19 | 0.9527 ± 0.0271 | 0.008075 ± 0.004639 |
|  |  |  | Katanda | 107 | 39 | 39 | 0.8919 ± 0.0210 | 0.006934 ± 0.003987 |
|  |  |  | Kanyamansira | 38 | 21 | 21 | 0.9403 ± 0.0232 | 0.007966 ± 0.004566 |
|  | cytb | Ndali-Kasenda | Nyabikere | 1 | 1 | 0 | 0.0000 ± 0.0000 | 0.000000 ± 0.000000 |
|  |  |  | Kitere | 10 | 4 | 6 | 0.7333 ± 0.1199 | 0.006332 ± 0.004242 |
|  |  |  | Wandakara | 1 | 1 | 0 | 0.0000 ± 0.0000 | 0.000000 ± 0.000000 |
|  |  |  | Kasenda | 8 | 4 | 5 | 0.7857 ± 0.1127 | 0.006502 ± 0.004465 |
|  |  |  | Mahuhura | 20 | 11 | 14 | 0.8684 ± 0.0570 | 0.008235 ± 0.004978 |
|  |  |  | Nyamirima | 5 | 3 | 4 | 0.8000 ± 0.1640 | 0.005805 ± 0.004474 |
|  |  |  | Rukwanzi | 13 | 6 | 7 | 0.7821 ± 0.1045 | 0.005751 ± 0.003823 |
|  |  |  | Katanda | 55 | 15 | 17 | 0.8222 ± 0.0338 | 0.006528 ± 0.003973 |
|  |  |  | Kanyamansira | 15 | 9 | 12 | 0.8857 ± 0.0686 | 0.007790 ± 0.004841 |
| *Echinoparyphium* sp. | COI2 | Bunyaruguru | Nyungu | 2 | 1 | 0 | 0.0000 ± 0.0000 | 0.000000 ± 0.000000 |
|  |  | Ndali-Kasenda | Nyabikere | 4 | 2 | 4 | 0.6667 ± 0.2041 | 0.007890 ± 0.006283 |
|  |  |  | Kasenda | 1 | 1 | 0 | 0.0000 ± 0.0000 | 0.000000 ± 0.000000 |
|  |  |  | Kanyamansira | 2 | 2 | 1 | 1.0000 ± 0.5000 | 0.002959 ± 0.004184 |
|  | NAD1 | Bunyaruguru | Nyungu | 4 | 2 | 9 | 0.5000 ± 0.2652 | 0.009574 ± 0.007097 |
|  |  |  | Maturo | 38 | 6 | 6 | 0.5861 ± 0.0746 | 0.002609 ± 0.001884 |
|  |  | Ndali-Kasenda | Nyabikere | 44 | 9 | 12 | 0.7008 ± 0.0671 | 0.007759 ± 0.004448 |
|  |  |  | Nyamirima | 3 | 3 | 10 | 1.0000 ± 0.2722 | 0.014184 ± 0.011485 |
|  |  |  | Kasenda | 13 | 5 | 11 | 0.6282 ± 0.1431 | 0.009547 ± 0.005650 |
|  |  |  | Kanyamansira | 7 | 4 | 9 | 0.8095 ± 0.1298 | 0.010132 ± 0.006452 |
|  | cytb | Bunyaruguru | Nyungu | 4 | 1 | 0 | 0.0000 ± 0.0000 | 0.000000 ± 0.000000 |
|  |  |  | Maturo | 50 | 2 | 1 | 0.0400 ± 0.0380 | 0.000106 ± 0.000315 |
|  |  | Ndali-Kasenda | Nyabikere | 39 | 1 | 0 | 0.0000 ± 0.0000 | 0.000000 ± 0.000000 |
|  |  |  | Nyamirima | 3 | 2 | 1 | 0.6667 ± 0.3143 | 0.001759 ± 0.002194 |
|  |  |  | Kasenda | 13 | 2 | 2 | 0.1538 ± 0.1261 | 0.000812 ± 0.001003 |
|  |  |  | Kanyamansira | 7 | 2 | 1 | 0.2857 ± 0.1964 | 0.000754 ± 0.001030 |
| Plagiorchioidea sp. | NAD1 | Bunyaruguru | Nyungu | 7 | 1 | 0 | 0.0000 ± 0.0000 | 0.000000 ± 0.000000 |
|  |  |  | Kigezi | 1 | 1 | 0 | 0.0000 ± 0.0000 | 0.000000 ± 0.000000 |
|  |  |  | Mirambi | 18 | 1 | 0 | 0.0000 ± 0.0000 | 0.000000 ± 0.000000 |
|  |  |  | Maturo | 190 | 1 | 0 | 0.0000 ± 0.0000 | 0.000000 ± 0.000000 |
|  |  | Ndali-Kasenda | Kasenda | 1 | 1 | 0 | 0.0000 ± 0.0000 | 0.000000 ± 0.000000 |
|  | cytb | Bunyaruguru | Nyungu | 4 | 1 | 0 | 0.0000 ± 0.0000 | 0.000000 ± 0.000000 |
|  |  |  | Kigezi | 2 | 1 | 0 | 0.0000 ± 0.0000 | 0.000000 ± 0.000000 |
|  |  |  | Mirambi | 15 | 1 | 0 | 0.0000 ± 0.0000 | 0.000000 ± 0.000000 |
|  |  |  | Maturo | 189 | 1 | 0 | 0.0000 ± 0.0000 | 0.000000 ± 0.000000 |

*Table S4: P-values obtained from the pairwise Wilcoxon tests with Hommel’s corrections to compare haplotype and nucleotide diversity between analysed markers for each species. Populations of Plagiorchioidea sp. were not included as no genetic polymorphism was observed within or among these populations. Populations with sample size <5 are excluded from these analyses. Values in bold indicate significant differences. Cells containing – indicate that no comparison was performed because the markers could not be amplified.*

|  |  | Haplotype diversity | | | Nucleotide diversity | | |
| --- | --- | --- | --- | --- | --- | --- | --- |
|  |  | COI1 | COI2 | NAD1 | COI1 | COI2 | NAD1 |
| *Bulinus tropicus* | COI2 | 0.65 |  |  | 0.88 |  |  |
|  | NAD1 | 0.59 | 0.59 |  | 0.88 | 0.88 |  |
| *Petasiger* sp. | COI2 | 0.77 |  |  | 0.43 |  |  |
|  | NAD1 | 0.43 | 0.59 |  | 0.25 | 0.95 |  |
|  | cytb | 0.77 | 0.77 | 0.28 | 0.43 | 0.95 | 0.95 |
| *Echinoparyphium* sp. | cytb | *–* | *–* | **0.03** | *–* | *–* | **0.03** |

*Table S5: P-values obtained from the pairwise Wilcoxon tests with Hommel’s corrections to compare haplotype and nucleotide diversity between the species analysed for each marker sequenced. Populations of Plagiorchioidea sp. were not included as no genetic polymorphism was observed within or among these populations. Populations with sample size <5 are excluded from these analyses. Values in bold indicate significant differences.*

|  |  | Haplotype diversity | | | Nucleotide diversity | |
| --- | --- | --- | --- | --- | --- | --- |
|  |  | *Petasiger* sp. | *Echinoparyphium* sp. | *Petasiger* sp. | | *Echinoparyphium* sp. |
| COI1 | *Bulinus tropicus* | **< 0.01** |  | **0.05** | |  |
| COI2 | *Bulinus tropicus* | **< 0.01** |  | **< 0.01** | |  |
| NAD1 | *Bulinus tropicus* | **< 0.01** | **0.04** | **< 0.01** | | **0.02** |
|  | *Echinoparyphium* sp. | **0.04** |  | 0.5522 | |  |
| cytb | *Echinoparyphium* sp. | **0.01** |  | **0.01** | |  |

*Table S6: Tajima’s* D *and Fu’s* Fs *tests of neutral evolution. Lakes with low sample size (<5 are excluded) and lakes with only 1 haplotype are indicated with –. Significance of p-value is signalled by * if < 0.05 for Tajima’s D or < 0.02 for Fu’s Fs and ** if < 0.001 (both tests).*

| Species | Lake | COI1 | | COI2 | | NAD1 | | | cytb | | |
| --- | --- | --- | --- | --- | --- | --- | --- | --- | --- | --- | --- |
|  |  | Tajima's *D* | Fu's *Fs* | Tajima's *D* | Fu's *Fs* | Tajima's *D* | Fu's *Fs* | Tajima's *D* | | Fu's *Fs* |  |
| *Bulinus tropicus* | Nyungu | -2.040* | 2.471 | -1.983* | 2.119 | -2.014* | 2.565 | - | | - |  |
|  | Maturo | 1.155 | 4.137 | 1.399 | 4.988 | 2.184 | 8.950 | - | | - |  |
|  | Kigezi | - | - | - | - | - | - | - | | - |  |
|  | Mirambi | 2.150 | 8.861 | 2.118 | 5.437 | 2.212 | 9.841 | - | | - |  |
|  | Nyabikere | 1.373 | 1.577 | -1069 | -1.906 | -1.069 | -1.906 | - | | - |  |
|  | Kitere | 0.268 | 0.296 | -0.507 | -0.470 | -0.374 | -0.791 | - | | - |  |
|  | Wandakara | -1.096 | -1.096 | -0.072 | -0.491 | 1.166 | 1.215 | - | | - |  |
|  | Kasenda | -0.705 | -1.749 | -1.338* | -4.487** | -0.993 | -2.485* | - | | - |  |
|  | Njarayabana | -0.058 | -1.602 | -0.503 | -1.657 | -1.070 | -4.424 | - | | - |  |
|  | Mahuhura | -0.461 | -1.331 | -2.087* | -10.732** | -2.283** | -12.148** | - | | - |  |
|  | Nyamirima | -1.358 | -0.237 | -0.275 | -0.438 | - | - | - | | - |  |
|  | Rukwanzi | 0.671 | 1.357 | 1.420 | 2.650 | 2.024 | 5.160 | - | | - |  |
|  | Nyinambuga | 1.356 | 2.328 | 1.356 | 2.328 | 1.633 | 4.240 | - | | - |  |
|  | Katanda | -1.197 | 0.606 | -0.769 | -0.855 | -1.517* | -0.297 | - | | - |  |
|  | Kanyamansira | -0.483 | -0.073 | -0.466 | -0.047 | -1.140 | -1.290 | - | | - |  |
| Petasiger sp. | Nyabikere | - | - | - | - | -0.516 | -2.855 | - | | - |  |
|  | Kitere | -0.191 | -0.445 | -0.991 | -1.381 | -1.262 | 0.482 | 0.544 | | 1.007 |  |
|  | Kasenda | -0.534 | -3.253 | -1.478 | -6.694** | -1.436 | -3.369 | 1.260 | | 0.583 |  |
|  | Mahuhura | - | - | -0.365 | -3.201 | -1.698* | -17.584** | -0.765 | | -3.702 |  |
|  | Nyamirima | - | - | 0.286 | -0.332 | -0.297 | -11.630** | 0.957 | | 0.804 |  |
|  | Rukwanzi | -1.798* | -1.113 | -0.192 | -1.825 | -0.644 | -17.133** | -0.128 | | -0.912 |  |
|  | Katanda | -1.332 | -3.752* | -1.529* | -10.073** | -1.739* | -26.260** | -1.028 | | -5.058 |  |
|  | Kanyamansira | - | - | -0.976 | -2.080 | -0.842 | -11.522** | -0.779 | | -2.878 |  |
| *Echinopariphium* sp. | Maturo | - | - | - | - | -0.382 | -0.741 | -1.103 | | -1.636 |  |
|  | Nyabikere | - | - | - | - | 0.974 | 0.862 | - | | - |  |
|  | Kasenda | - | - | - | - | 1.075 | 2.112 | -1.468 | | 0.362 |  |
|  | Kanyamansira | - | - | - | - | 1.575 | 1.621 | -1.006 | | -0.095 |  |
| Plagiorchioidea sp. | Rukwanzi | - | - | - | - | - | - | - | | - |  |
|  | Katanda | - | - | - | - | - | - | - | | - |  |
|  | Kanyamansira | - | - | - | - | - | - | - | | - |  |

*Table S7: Results of Mantel tests to examine correlations between Slatkin’s genetic distance among populations and the Euclidian geographic distance between the lakes where these populations were sampled.*

| Organism | Marker | Correlation coefficient | Variation explained | p-value |
| --- | --- | --- | --- | --- |
| *Bulinus tropicus* | Multi-locus (n = 15) | 0.199 | 4.0 % | 0.052 |
|  | COI1 (n = 15) | 0.230 | 5.3 % | <0.050 |
|  | COI2 (n = 15) | 0.199 | 3.9 % | <0.050 |
|  | NAD1 (n = 15) | 0.065 | 0.4 % | >0.100 |
| *Petasiger* sp. | COI1 (n = 4) | 0.491 | 24.1 % | >0.100 |
|  | COI2 (n = 7) | -0.602 | 36.2 % | >0.100 |
|  | cytb (n = 7) | -0.195 | 3.8 % | >0.100 |
|  | NAD1 (n = 8) | 0.161 | 2.6 % | >0.100 |
| *Echinoparyphium* sp. | cytb (n = 4) | -244 | 5.9 % | >0.100 |
|  | NAD1 (n = 4) | 0.731 | 53.5 % | >0.100 |

*Figure S1: TCS haplotype networks of: A. COI1 marker of* Bulinus tropicus *(n = 823); B. COI2 marker of* Bulinus tropicus *(n = 812); C. NAD1 marker of* B. tropicus *(n = 816); D. COI1 of* Petasiger *sp. (n = 71); ); E. COI2 of* Petasiger *sp. (n = 171); ); F. cytb of* Petasiger *sp. (n = 128); G. cytb of* Echinoparyphium *sp. (n = 116). Each circle represents a unique haplotype, circle size being proportional to the number of specimens with the haplotype/ Colors proportions in the circles provides information on the population of the specimens for each population (blue colors = populations from the Bunyaruguru cluster, red to yellow colors = populations from the Ndali-Kasenda cluster). Dashes on segments connecting haplotype circles represent the number of inferred mutation steps between haplotypes.*

*Figure S2 Relationships between Slatkin’s genetic distance among populations and the geographic distance between the lakes where the populations were sampled for* Bulinus tropicus *(multi-locus dataset, 15 lakes),* Petasiger *sp. (NAD1 marker, eight lakes) and* Echinoparyphium *sp. (NAD1 marker, four lakes). A linear regression line is fitted for illustration in the plot of* Bulinus tropicus *as a near-significant isolation-by-distance pattern was observed using a Mantel test (p-value = 0.052). Geographic distances were log10-transformed in the* B. tropicus *plot to increase visual clarity.*
